# Maternal Health in Crisis: A Scoping Review of Barriers and Facilitators to Safe Abortion Care in Humanitarian Crises

**DOI:** 10.3389/fgwh.2021.699121

**Published:** 2021-09-21

**Authors:** Bianca Dias Amaral, Dikaios Sakellariou

**Affiliations:** ^1^London School of Hygiene and Tropical Medicine, London, United Kingdom; ^2^School of Healthcare Sciences, Cardiff University, Cardiff, United Kingdom; ^3^European University Cyprus, Nicosia, Cyprus

**Keywords:** abortion, humanitarian crises, maternal health, sexual and reproductive health, global gag rule

## Abstract

During humanitarian crises, women are particularly vulnerable to unwanted pregnancy. Unsafe abortion is among the five leading causes of maternal mortality and it is the only one which is entirely preventable. This study aimed to identify the barriers and facilitators to the provision of safe abortion care by humanitarian organisations. We performed a scoping review of the literature in July 2020, covering the years 2010–2020, on the following databases: Medline, Global Health, CINAHL Plus and ReliefWeb. We critically appraised all included articles and we conducted a narrative synthesis of the findings. We retrieved 881 articles. After removing duplicates and excluding articles that did not meet the inclusion criteria, twenty-four articles published between 2015 and 2020 were included in the review. Nine of the included papers were non-research practise items. The findings revealed five main themes: legal environment; context; stigma; economic factors; and service delivery. Restrictive laws, stigma, and lack of funding were reported as the main barriers to safe abortion, while the main facilitators were the fact that abortion is permitted under some circumstances in most countries, humanitarian actors' ability to inform healthcare policies at the onset of a humanitarian crisis, and community engagement. This scoping review revealed a dearth of published research. Increased dissemination of studies on Termination of Pregnancy (ToP) could increase the visibility of unsafe abortion and the need to provide ToP in humanitarian settings. Moreover, humanitarian organisations need to have a clear protocol on safe abortion and an in-depth understanding of relevant legislation, including the International Humanitarian Law, in order to provide this service to the full extent of the law.

## Introduction

Humanitarian crises such as conflicts, epidemics and natural disasters threaten the safety, security, health, and wellbeing of the population where they take place and can lead to compromised availability and suboptimal delivery of sexual and reproductive health (SRH) care, (1) and, even where such services are provided, to low utilization (2). Humanitarian crises disproportionately affect the health of women by reducing their access to SRH services and exposing them to sexual violence (3). The UN Committee on the Elimination of Discrimination Against Women stresses that:

*“States parties should ensure that measures are taken to prevent coercion in regard to fertility and reproduction, and to ensure that women are not forced to seek unsafe medical procedures such as illegal abortion because of lack of appropriate services in regard to fertility control* [(4), p.289].”

Despite significant positive changes in the last ten years, ToP through induced abortion is rarely included in the agenda of organisations providing SRH care in humanitarian settings (5). Induced abortion is among the safest medical procedures and mid-level healthcare personnel without sophisticated infrastructure can safely perform it in a primary healthcare setting (5). In fact, ToP provision requires the same kind of personnel and nearly the same kind of medical supply as the provision of Post-Abortion Care (PAC). Safe Abortion Care (SAC) incorporates both PAC and ToP. ToP is the induction of abortion and PAC is the care provided to women with an ongoing abortion, whether it is an involuntary loss of pregnancy or an incomplete induced abortion. As it is often impossible to verify whether an incomplete abortion is the result of a miscarriage or a voluntary termination of the pregnancy, women might report having a miscarriage when they are going through a ToP complication, especially where ToP is stigmatised and forbidden by law. Access to safe abortions is a fundamental human right and yet many women still do not have access to this healthcare service, especially in humanitarian settings, (6) and they are forced to resort to unsafe abortions, (7) with serious consequences to their health (8, 9).

The United Nations (UN) Sustainable Development Goals (SDGs) make explicit reference both to maternal health and to the provision of SRH care. SDG No. 3.1 commits to reducing “the global maternal mortality ratio to less than 70 per 100,000 live births” by 2030, while SDGs No. 3.7 and No. 5.6 aim to ensure universal coverage of SRH services (10). Unsafe abortion, which is the termination of pregnancy (ToP) performed by unskilled personnel, in unsterile conditions, and/or through the use of dangerous techniques (11) accounts for life-long disabilities such as sterilisation, infections, and 7.9% of all maternal deaths worldwide, and is the only cause of maternal death that is completely avoidable (12).

Reproductive healthcare was greatly neglected by humanitarian organisations until the 1990s. In 1994, the UN organised the International Conference on Population and Development in Cairo, which recognised reproductive health as a basic human right (13). Two years later, the Minimum Initial Service Package (MISP) for Sexual and Reproductive Health in Crisis Situations, a series of essential actions required to respond to reproductive health needs at the onset of a humanitarian crisis, was included in the initial field-test version of the *Reproductive Health in Refugee Situations: An Inter-Agency Field Manual* (hereafter field manual), (14) and in 2004 it became part of the Sphere Minimum Standards, a set of essential standards in humanitarian response (15). Subsequent revisions of the inter-agency field manual in 2010 (16) and 2018 (17) discussed safe abortion care as an important preventive measure against maternal morbidity and mortality in humanitarian settings, while the 2018 revision of the MISP added safe abortion as a specific priority action (18).

Non- and intergovernmental humanitarian organisations are essential for the provision of humanitarian medical care in contexts such as natural disasters, disease epidemics, and conflict-affected countries, where the local health system no longer has the capacity to timely respond to the health needs of the population. However, providing ToP might not be included in the agenda of non- and intergovernmental organisations for several reasons, including unrecognition of the need of ToP in comparison to other life-saving services such as food, water and sanitation; the technical “complications” regarding its provision, which requires specific protocols and medications; its legality in the country of operations, considering that the majority of countries restricts termination of pregnancy to certain circumstances; and the unwillingness of major donors to fund ToP. Many of these assumptions have however been shown to be incorrect. Firstly, usafe abortion is among the leading causes of maternal mortality, which is expected to increase in humanitarian crises due to the collapse of healthcare services. Secondly, SAC can be performed by nurses and midwives in health centres and it does not require sophisticated materials. Thirdly, abortion is permitted under some circumstances in the vast majority of countries and is totally forbidden only in six of them. Lastly, although important donors such as the US do not fund abortion-related services, many other countries do so, including several European countries (5).

Currently, there seems to be an important gap both in the provision of ToP in humanitarian crises and on relevant published research, adding to the invisibility of this important public health issue (19). This scoping review aimed to identify the barriers and facilitators to the provision of safe abortion care by humanitarian organisations. The study protocol has not been registered.

## Methods

We conducted a scoping review of the literature in July 2020, seeking to answer the question: “What are the challenges humanitarian organisations face in providing ToP in humanitarian settings and how do they overcome them?” We used the PRISMA-ScR (Preferred Reporting Items for Systematic reviews and Meta-Analyses extension for Scoping Reviews) guidelines (20) to report the review process. The eligibility criteria are presented in Table 1.

**Table 1:** Eligibility criteria.

**Inclusion criteria**	**Exclusion criteria**
Articles reporting on ToP in humanitarian settings in low and middle-income countries.	Articles on ToP interventions provided solely by services under local ministries of health without the participation of humanitarian organisations.
Articles published from 2010 until 2020, because 2010 was the year a chapter on abortion care was added to the inter-agency field manual on reproductive health in humanitarian settings (16).	Articles reporting solely on post-abortion care as those include involuntary loss of pregnancy, and the focus of this study is to investigate the literature regarding access to termination of pregnancy.
Articles published in English, Spanish, or Portuguese, as those are the only languages familiar to the authors.	Articles reporting on ToP in high-income countries were excluded as the vast majority of humanitarian emergencies and maternal mortality occur in low and middle-income countries.
All research designs and practise literature.	

### Information Sources and Search

We searched the following databases: Medline, Global Health, CINAHL Plus, and ReliefWeb. Selected journals such as *Conflict and Health*, and *Health and Human Rights* were also hand-searched due to their relevance. The search strategy for each database can be found in Supplementary Material A. The terms were combined using a Boolean OR operator where possible. Since all articles had an English abstract, there was not a need for Spanish and Portuguese search terms. The reference lists of all relevant articles were also hand-searched for other possible articles that did not appear during the literature search. References were stored in the citation software Zotero and a copy was kept in a separate excel sheet.

### Definition of Terms

*Humanitarian organisations/Non-governmental organisations (NGOs)*: these terms are used interchangeably to refer to “legally-constituted association(s), operating independently of governments, with a social or socio-political agenda, and not conventionally for profit” (3). This study also included UN agencies in its scope, which are intergovernmental organisations (IGO) aiming to render humanitarian aid, such as the United Nations Population Fund (UNFPA), specialised in the provision of sexual and reproductive health care, and the United Nations High Commissioner for Refugees (UNHCR). UN agencies operate through an agreement between member states under international law and are established and controlled by governments.

*Humanitarian setting:* humanitarian crisis contexts such as conflict-affected countries; communities hosting internally displaced persons and refugees; epidemics; and natural disasters, where humanitarian organisations work to relieve human suffering, “especially when there is an actual or imminent threat to life, health, subsistence or security” (3).

*Termination of pregnancy (on request):* the medical procedure of induced abortion, either medically (by drugs), or surgically (by manula or electric aspiration, or dilation and evacuation) (21).

### Screening and Data Extraction

The first author initially screened articles based on title, keywords, and abstract. When it was not clear whether the eligibility criteria were met, both authors reviewed the full article to determine if it met the inclusion criteria and none of the exclusion ones presented in Table 1. There was no disagreement between the two reviewers in this process. Full text was accessed for all included articles. Guided by the study aim, the first author extracted bibliographical data and information on barriers and facilitators to safe abortion care implementation by humanitarian organisations, participants, strengths and weaknesses for each included source and entered them into an excel table for analysis, while the second author independently checked all included articles, noting down the main findings, which concurred with those identified by the first author. All included sources were critically appraised.

### Critical Appraisal

We used the Mixed Methods Appraisal Tool (MMAT) (see Supplementary Material C), to critically appraise research articles following a qualitative, quantitative, or mixed methods design, and the Aveyard et al. (22) generic appraisal tool, to appraise practise literature (see Supplementary Material D). The CASP (Critical Appraisal Skills Programme) checklist tool was used to review systematic reviews (see Supplementary Material E). Critically appraising the included sources enabled us to identify some of their weaknesses, which included: selection bias; (23, 24) reporting bias due to a lack of a systematic approach in conducting surveys; (25) limited engagement with available literature; (26) and recall bias of interviews being conducted ten years after phenomenon being investigated (27). It is important to note however that conducting research in acute and emergency humanitarian crises is challenging mostly due to insecurity and population move, as well as the often poor surveillance due to the prioritisation of lifesaving activities. Surveillance data raised by prospective research, which is what is often funded and published, remains a particular challenge as the future of the research subjects (persons) is unknown and they might not be traceable until the end of the study.

### Synthesis of Results

Following critical appraisal, we used a process of narrative synthesis to discuss the findings of the articles included in the review (28). This allowed the synthesis of results from a variety of sources, including quantitative and qualitative studies, systematic reviews, and non-research practise literature items, such as commentaries and reports. We first read the findings and discussion sections (and other sections, where findings were presented) of the included articles and generated codes, with a focus on facilitators and barriers to access to ToP, guided by the review aim. These codes were descriptive and illustrated the main issues reported in the dataset. These codes were then synthesised into themes, through an iterative process where ongoing analysis contributed to the development of themes, which then in turn informed analysis.

## Results

**Figure 1:**
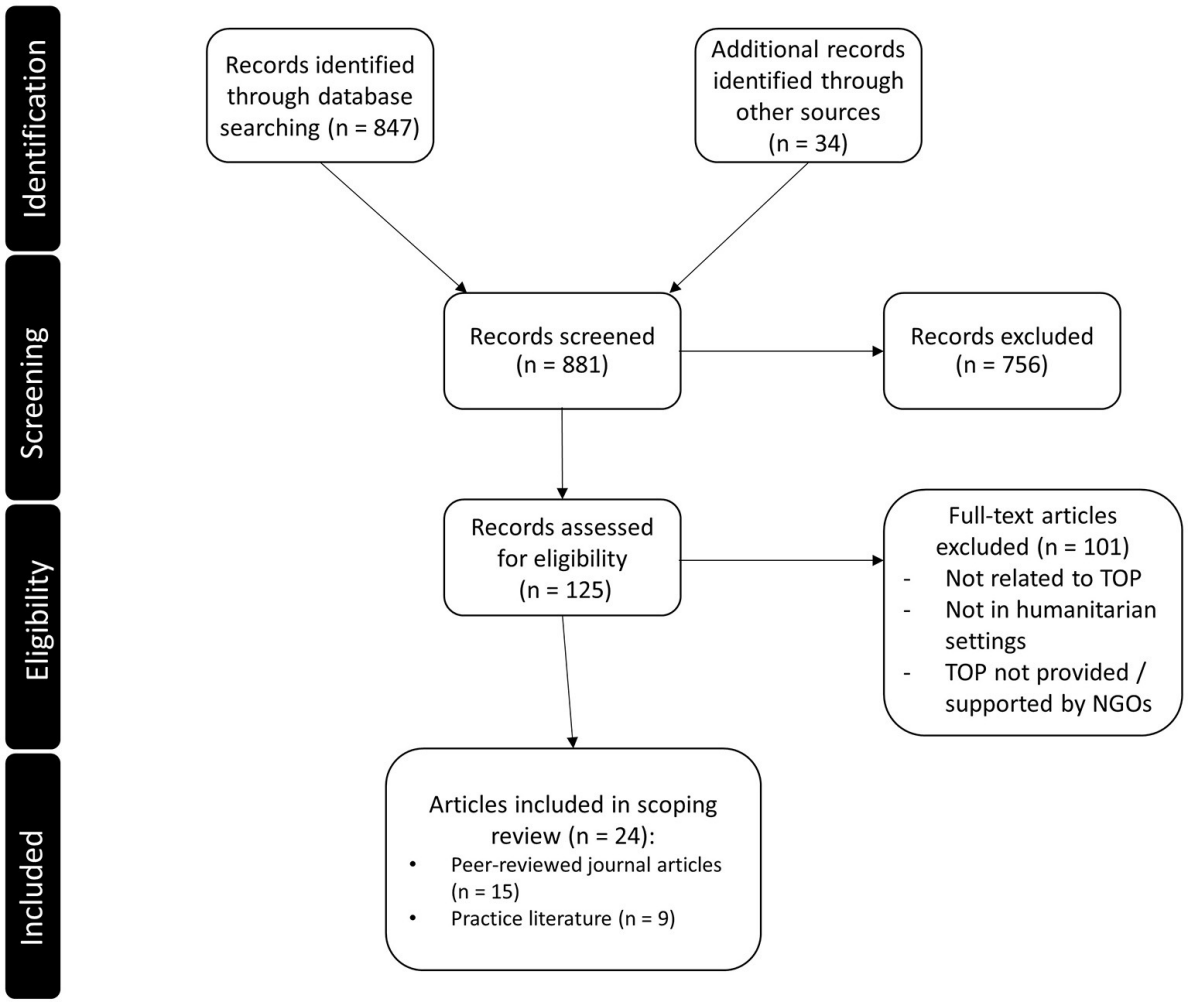
PRISMA diagram.

In this review, we included twenty-four articles (research studies and non-research practise literature in the form of reports, commentaries, and opinion pieces) published between 2015 and 2020. We found no eligible articles published between 2010 and 2014. See Figure 1 for information about numbers of sources at each stage of the review process. Fifteen articles were classified as research and nine as non-research, that is, studies without a clear description of its methodology, and they were all written in English (see Table 2). See Supplementary Material B for the complete table of the included articles.

**Table 2:** Included article characteristics.

**Type of study**	
Qualitative	*n* = 7
Quantitative	*n* = 3
Mixed-methods	*n* = 4
Systematic review	*n* = 1
Non-research practise literature (expert opinion, interview, reports etc.)	*n* = 9
**Geographical focus** ^*^	
Global	*n* = 14
Democratic Republic of Congo (DRC)	*n* = 3
Burkina Faso	*n* = 1
Colombia	*n* = 1
Jordan	*n* = 1
Myanmar	*n* = 1
Nepal	*n* = 1
Sudan	*n* = 2
Occupied Palestinian Territories	*n* = 1
Uganda	*n* = 1

* *Some articles referred to more than one geographical region*.

### Narrative Synthesis

Following critical appraisal, all articles were examined to identify the main barriers faced by humanitarian organisations regarding the provision of safe abortion care in their programmes, as well as the mitigation measures utilised to overcome these barriers. The findings revealed five main themes in relation to ToP implementation: legal environment; context; stigma; economic factors; and service delivery. Barriers and facilitators to access ToP were collated under each theme, revealing both challenges and mitigation strategies for each theme.

### Legal Environment

Most articles referred to legal restrictions as one of the main barriers to access ToP, and the main reason why humanitarian organisations may opt not to offer ToP under any circumstances in humanitarian contexts (5, 23, 24, 27–35). Among other reasons, restrictive abortion laws (23, 24, 29, 30, 34, 35), i.e. laws that restrict women's access to ToP services, and organisations' lack of legal knowledge about ToP provision in the country of operations (5, 29, 33, 34) make ToP inaccessible to women within the legal framework (27, 29, 31, 32).

The inconsistent interpretation of the criminal code, laws and policies by the authorities, (32) and the difficulty to navigate through legal restrictions (33) (these two last reasons were identified in practise literature), may encourage healthcare services such as MoH facilities to impose restrictive hospital ToP policies (24). Also, displaced women in regions where ToP is not permitted might be forced to travel to another country, where ToP is legal, and face catastrophic expenditures as a consequence of the out-of-pocket payments (24).

The global gag rule was introduced by the administration of US President Ronald Reagan in 1984, and has since been implemented by Republican administrations and repealed by each Democrat administration. Until 2017, the global gag rule stipulated that for non-US NGOs (the rule does not apply to US NGOs) to receive US government family planning funding, they could not perform or actively promote abortion as a contraceptive method, even though such activities are paid for with NGO's non-USAID funds.

Introduced in 2017, the Protecting Life in Global Health Assistance (PLGHA) policy expanded restrictions to organisations receiving US government global health funding (36). It must be noted that while neither the pre- nor the post-2017 version of the policy applies to US-government funded humanitarian assistance, many organisations receive both US government funded humanitarian and global health assistance, making them subject to restrictions.

The main legal facilitator is the fact that ToP is permitted under certain circumstances in most countries (5, 24, 31). In conflict and post-conflict contexts, International Humanitarian Law overrules the local laws (5, 32). According to International Humanitarian Law, pregnant women's needs have to be considered, including their right to healthcare access, while healthcare staff who provide ToP need to be protected from local prosecution (32).

Additionally, despite US restrictions on the use of funding, the USA Leahy Amendment permits counselling and information about all pregnancy options in line with local laws, even by those organisations receiving US funds (32).

Moreover, all countries permit post-abortion care. In Nepal, where an abortion law was passed in 2006, one of the main facilitators to the provision of safe abortion care was the fact that port-abortion care was already widely provided and providers had the necessary skills and equipment (15).

#### Context

Immediate consequences of humanitarian crises, such as health system collapse, (35) insecurity (37), travel restrictions, (24, 34) and sexual violence (37) complicate access to and delivery of healthcare services. Additionally, logistical challenges such as the mountainous terrain of Nepal, (15) or checkpoints in Colombia, (30) can impose an extra obstacle to accessing care.

Barriers to ToP also include possible tension with local authorities and weak communication between stakeholders (15, 33). Lastly, one commentary reported that including safe abortion in the MISP might put field staff, patients and operations at risk, since negotiations with local authorities often take long time (38). Because the MISP entails providing SRH services within 48 hours of the onset of a humanitarian crisis, including safe abortion so early in the implementation of humanitarian activities would mean no time to properly conduct a risk analysis before the provision of safe abortion, potentially leading to an abrupt termination of the local activities of international NGOs.

Political opportunity window was the most cited contextual factor facilitating the access to ToP (23, 29, 37). During humanitarian crises, international organisations might be in a position to inform healthcare policies and regulations, opening the opportunity to designing safe abortion care services. Furthermore, identifying an agency within the health sector to coordinate the provision of SRH, objective 1 of the MISP, (25) with support from the ministry of health (29) was recognised as a facilitator. A pre-established Memorandum of Understanding between the ministry and humanitarian organisations can enable fast transitioning into emergency response when needed, allowing the rapid establishment of national-level coordination of the emergency response (15). On the community level, raising awareness with local authorities, (37) as well as engaging with community leaders to become agents of change have shown to facilitate access to abortion in the DRC and other countries (39).

#### Stigma

Social stigma was an important barrier in accessing termination of pregnancy (15, 23, 24, 29, 34–37, 40). Fear of social repercussions such as spousal abandonment (41) also affected the accessibility of ToP. Religious and cultural beliefs against ToP, (31) nested within conservative and patriarchal power structures, (31) played an important role in restricting the provision of and access to ToP (31, 33). An important negative structural factor related to ToP access was the politicisation of childbirth, in which pro-natalist policies and traditions affect the women's decisions regarding reproduction (23, 24, 39).

On the community level, cultural attitudes and beliefs can also hamper the access to ToP, for example, when there is a requirement of approval by the woman's husband, as it is the case in South Sudan and the Occupied Palestinian Territories (23, 24, 40). Both research and practise literature mentioned conscientious objection, i.e. the right of providers to refuse performing ToP, as a barrier for women seeking abortion in Colombia and elsewhere, even when ToP can be legally provided (29, 33). In some contexts such as in Jordan, the lack of female SRH staff was reported as a barrier to accessing ToP (25).

Lasly, due to the stigma related to the provision of termination of pregnancy, which might involve security risks for its personnel and/or reduction of fundings, organisations that run program evaluations might exclude the provision of safe abortion care from evaluation reports (42–44). This exacerbates the invisibility of ToP and makes it harder to advocate and get funds for and implement such activities in humanitarian settings (35).

A facilitating factor described in the reviewed literature related to informal healthcare networks, enabling women to have access to abortion pills, herbs, and getting information on ToP by their friends and relatives (23). Furthermore, training workshops for healthcare professionals, such as the Values Clarification Workshop, (23) and actively reaching out to staff to create an environment in which personal feelings towards abortion can co-exist the professional responsibility, as identified in practise literature (33), were also mentioned as facilitating factors. In South Sudan, providing ToP in secret by private or NGO clinics was considered an individual-level harm-reduction approach to violence from male partners (23).

#### Economic Factors

The lack of funding fo abortion services was the main economic barrier related to providing safe abortion care, as reported by several sources, both research and practise literature (5, 15, 24–26, 32, 34, 40, 41, 45). However, it is important to note that funding for SRH services in general is also a challenge and often SRH programs are under-funded. The allocation of funds for ToP within the SRH budget is an additional challenge inside the humanitarian organisations. As the majority of facilities in humanitarian crises is partly or entirely funded by NGOs, restricting specific funds translates into restricting specific services, in this case, safe abortion care.

In South Sudan, internal financial difficulties in NGOs and opposition of donors to ToP were also reported as barriers to the provision of safe abortion (23). The exclusion and invisibility of abortion care from SRH proposals also contributes to the lack of funding of such programs (46). NGOs might sometimes request unespecific SRH funds for donors, as a way to enhance the chances of ToP services being funded under the SRH umbrella. If this tactic could be effective in ensuring funds opposed to donors' interests, it thus contributes to maintaining the invisibility of the need to provide termination of pregnancy in humanitarian settings as a way to prevent maternal mortality (46).

The high costs related to accessing ToP in private clinics, including travel and transportation costs, were considered an important economic barrier in accessing ToP in Nepal, Uganda, the Occupied Palestinian Territories, Thailand and the DRC (15, 24, 30, 34, 35). The availability of SRH funding in humanitarian projects, as it was described in Zaatari refugee camp in Jordan was a facilitating factor (25). Also, the resource mobilisation after the earthquake in Nepal, (15) and the financial support provided to Burmese refugee women in Thailand, including coverage of the cost of ToP and travel expenses, enabled women to seek safe termination of pregnancy (34).

Some of the economic facilitating factors mentioned (mostly in the practise literature) included the increase in SRH funding in humanitarian settings over time, (5, 41, 46) the segregation of non-US humanitarian aid from US donors to keep it free from restrictions, (32) and seeking non-US funding options for ToP, especially in the European Union (5).

#### Structural Issues Related to Service Delivery

##### Quality of ToP Services

Issues pertaining to service provision were among the main barriers to accessing ToP. The included articles showed low trust of ToP providers by the community, (15, 23, 24) a perceived low quality of SRH services offered, (25) inadequate staffing (15, 33–35, 39) and inadequately trained staff (15, 31). In Burkina Faso, South Sudan, and in the DRC, structural barriers associated with lack of training and lack of equipment were further amplified due to stigma related to ToP, so even when women had a miscarriage, they feared being judged and mistreated at the hospital (31). Finally, lack of information regarding SRH and safe abortion care (34) and the low awareness of service provision (15) can also be a barrier to safe abortion care, as reported by women in Jordan, Thailand, and the DRC (25, 34).

Regarding facilitators of access to ToP, established pre-crisis health services and a skilled workforce in Nepal and Jordan, trained in SRH, facilitated the delivery of ToP in these countries (15, 25). Additionally, the adoption of ToP protocols by NGOs and the sensitisation of medical staff to ensure quality of care and patient confidentiality were associated with successful ToP implementation programs in practise literature (33, 47).

##### Availability and Access to ToP

Inadequate infrastructure (24, 25, 27, 34) and lacking or destroyed supplies (15, 25, 31, 35) were among the main barriers to accessing ToP. In many humanitarian programs, even referrals to safe abortion care or to port-abortion care are not offered to women in need (27). In South Sudan, healthcare staff reported that providing ToP was a security risk, which in turn affected its provision (23, 33). Also, practise literature suggests that abortion providers may resent NGOs for providing free and safe care, taking business away from them (33). Humanitarian organisations appeared to be perceived as being responsible for some of the barriers to accessing ToP, due to several reasons. Firstly, the lack of appropriate needs assessments often leads to low awareness of local capacity and the duplication of activities (15). Secondly, there is a lack of ToP prioritisation in humanitarian programs, (5) and SRH needs, including abortion, are perceived as secondary during the onset of the crisis, as was the case after the earthquake in Nepal (15). Moreover, some NGOs are not allowed to work in countries such as South Sudan, (23) and, despite progressive policies, they still face internal and external resistance in different levels, as it is the case for Doctors Without Borders (MSF), (33) and might report ToP as a “complicated” activity to be implemented (5). For instance, NGO staff occupying leadership and decision-making positions who are personally against ToP, might not concentrate enough efforts to ensure ToP policy implementation in the field (29, 33). The lack of support from the local health system (24) and from the health cluster, adding to the lack or limited funding from donors (5, 15, 24–26, 32, 34, 40, 41, 45), pose yet another barrier to providing SAC in humanitarian crises.

A facilitator of ToP provision is the fact that it only needs basic infrastructure, it is not always dependent on electricity, and can be provided by nurses and midwives in health centres (5, 29). Additionally, a report suggested that international NGO staff providing ToP might be less exposed to consequences, as can be seen in this excerpt:

*“MSF (Médecins Sans Frontières) considers that national staff are particularly exposed to potential repercussions resulting from the provision of safe abortion care in their home country and community, and in places with legal restrictions it is MSF international medical staff who assume the responsibility of providing the necessary care* [(33), p.3].”

Furthermore, it was seen as important for NGOs to fully support all medical professionals providing ToP (33). Finally, the growth of SRH-related institutional capacity in humanitarian settings between 2004 and 2014 reported by the Inter-Agency Working Group (27) and the explicit inclusion of safe abortion care in the 2010 inter-agency field manual (47) can facilitate the implementation of safe abortion care services in emergencies.

## Discussion

Pregnant women in humanitarian settings who wish to terminate their pregnancy face numerous barriers. This study highlights barriers and facilitators to ToP provision in humanitarian contexts, relating to five main themes: legal environment, context, stigma, economic factors, and service delivery. Restrictive abortion laws, the broader funding context, resistance by NGOs, and stigma were the main barriers to ToP provision. A country may have ToP as legal on request, within a specific gestational period, with no other conditions for its provision, or have laws that restrict access to ToP, allowing it only under specific circumstances. This means that abortion is not provided upon request solely and needs further reasons to be performed, which can lead to barriers to access (48). The main facilitators were the fact that abortion is permitted under some circumstances in most countries, the ability of humanitarian actors to form political alliances to promote women's health, and community engagement, through, for example, informal networks and engaging with community leaders to raise awareness on ToP.

Findings also reveal other important and less explored barriers related to stigma, such as the abortion data exclusion from SRH evaluations of humanitarian NGO programmes (42–44) and from reproductive health funding proposals (41, 46). By including explicit data on termination of pregnancy in evaluation reports and funding proposals, NGOs might risk a reduction in their fundings from donors opposed to ToP and, in countries with highly restrictive abortion laws, NGOs might face increased security risks. The delay in including abortion care in obstetric policies and protocols of NGOs and in recognising it as part of the SRH essential services to be delivered in humanitarian settings, led to its further exclusion from academic research until very recently.

Out of the 24 articles included in the scoping review, only 15 were research studies, whereas nine belonged to practise literature, mainly in the form of opinion pieces and commentaries. Most research articles included in the study referred to people's perceptions towards ToP rather than the barriers within the organisations providing it. In this way, responsibility for access to ToP was shifted to women and their local contexts, with little exploration of the organisations providing, or not, such services.

### Study Limitations

Among the weaknesses of this study is that its methodology was restricted to published articles only, therefore it was not fairly representative of the reality faced by NGOs operating in emergency contexts, where TOP might be provided but a study on the subject not run or published due to the prioritisation of lifesaving activities.

Moreover, due to the limited resources, the authors had to narrow the scope of this review in order to finish it on time. For example, the decision to exclude PAC-focused articles was not ideal, as it is known that post-abortion care is the emergency treatment for complications resulting from spontaneous or induced abortion. This is an important limitation of this article, although one of its main purposes was to gather all the studies explicitly referring to ToP in humanitarian crises and how organisations have managed to overcome the many limitations curtailing its provision.

Excluding articles focusing solely on post-abortion care might have led to some relevant articles being missed, especially from contexts where women may not report a ToP attempt when arriving at a health facility with an incomplete abortion. To address possible publication bias, we included both research and practise literature, to ensure we capture a wide range of evidence. Furthermore, we performed the literature search on four databases and two journals and the duplication of articles indicated thorough coverage.

Another important limitation of this study is the fact that, during acute emergency humanitarian crises, there is a massive population movement, within or outside of the country, making it difficult to have consistent data, and often research is neither prioritised nor funded by donors, as the main objective of the operations is to quickly prevent deaths and not to report data. Lastly, abortion is highly stigmatised worldwide and, as an attempt not to conflict with donors' interests, organisations might decide not to publish studies regarding ToP, making the body of literature on this specific topic very limited.

This study highlights evidence suggesting that the provision of abortion services in humanitarian settings is directly impacted by the global gag rule (36, 49). Most organisations need to negotiate their priorities with their donors, particularly when activities are heavily subsidised by governmental bodies, and donors may not be willing to support ToP on request. The PLGHA policy (often referred to as the ‘global gag rule’, and known as the Mexico City Policy prior to 2017) is an example of how major donors can impact the delivery of SRH services in humanitarian settings (49, 50). As the US has become the main humanitarian donor worldwide, its decisions regarding ToP can have a big impact on access to and delivery of ToP.

PLGHA was quickly revoked in the United States when Joseph Biden assumed presidency in January 2021 and the scenario is optimistic for the coming years in relation to ToP. However, as soon as a republican president takes office in the US presidency, it is likely that humanitarian NGOs have to choose whether to provide ToP and reduce their funding, consequently reducing life-saving activities, or to receive global health funds from the US and no longer provide, refer or advocate women to ToP (51).

To revert NGO's lack of legal knowledge in regards to ToP provision in different countries of operations, (5, 29, 33, 34)investing in a local legal advisor could be an initial solution to better understand in which legal terms ToP can be safely provided. Induced abortion is permitted under some circumstances in 190 countries, often to preserve the physical and mental health of the woman and, in conflict-affected countries, the International Humanitarian Law overrules the local laws and, at least in theory, it ensures healthcare to all according to their needs, including ToP on request (32). It is also important for humanitarian organisation to have access to legal experts to address the implications of the global gag rule whenever the policy is re-invoked (52). The Protocol to the African Charter on Human and People's Rights on the Rights of Women in Africa, also known as the Maputo Protocol, the main legal instrument for the protection of the rights of women and girls in Africa, (53) is one of very few international instruments that specifically recognise access to safe and legal abortion as a human right. Several countries in Africa have signed and ratified it, which can be seen as the first step towards the provision of safe abortion services in that region (54).

## Recommendations

Safe abortion care must be included in evaluations of SRH programmes. Humanitarian organisations providing SRH services must have a clear protocol on safe abortion for their healthcare staff, ensuring its provision in the field level through project evaluation and auditing, as well as backing up the staff who provide ToP. Secondly, humanitarian organisations must have an in-depth understanding of the local laws regarding safe abortion care and a strong knowledge of the International Humanitarian Law when wishing to provide ToP in warzones and protracted crises. Thus, it is recommended that these humanitarian organisations make use of a local legal advisor so that termination of pregnancy can be provided to the full extent of the law in every country of operations.

Humanitarian organisations must have clear policies and guidelines so that their staff feel confident to provide safe abortion care within the legal framework as well as providing a safe space for staff to share their feelings about induced abortion, while ensuring staff abide to the organisation's policies. Safe abortion care must be integrated in the general SRH care in all humanitarian settings from the onset of the crisis and it is recommended that all staff involved in ToP provision go through a Values Clarification Workshop prior to the implementation of the service. Moreover, prior to implementing abortion services, stakeholder involvement through engagement with community leaders and local authorities are key to the success of the roll out of safe abortion care services. It is also essential to make good use of the political window lobbying strongly with the local MoH for a change in the healthcare provision and promote gender-sensitive services.

Governments with restricted abortion laws, especially those where induced abortion is not permitted under any circumstance, urgently need to review the impact of such practises in their maternal mortality rates, seeking to provide evidence-based care to all women in need. It is also the social responsibility of the society, in particular of women' groups and feminists' associations to fight for safe abortion care provision in their country. Additionally, it is also the role of the government to decriminalise ToP. In many countries, women coming from middle and high income households are able to access termination of pregnancy on request as long as a large sum of money is paid, in clinics that operate exclusively for that, despite the restrictive abortion laws, whereas women from low income families are obliged to seek unsafe abortion and are often stigmatised in public hospitals, if not sent to gaol. Governments of high income countries, responsible for much of the humanitarian aid funding, must also ensure that funding for safe abortion care is present.

Finally, as with any other type of healthcare services provided in humanitarian contexts, ToP must be offered free of charge, in privacy and with confidentiality. Organisations must ensure consistent earmarked SRH funds and ideally seek consistent, non-US-governmental donors, to avoid service disruption due to the global gag rule whenever the latter is invoked.

## Conclusion

This scoping review highlighted the lack of research on the provision of termination of pregnancy in humanitarian settings, with several of the included articles being commentaries, reports, and opinion pieces rather than research articles. The findings suggest that the main barriers to safe abortion implementation by organisations are restrictive abortion laws, lack of funding, and stigma. Among the most common facilitators to ToP are the fact that it is permitted under certain circumstances in almost every country, the political opportunity window at the onset of a humanitarian crisis allowing humanitarian organisations to promote ToP services, and community engagement. Unsafe abortion is among the leading causes of maternal mortality and it is the only one which is entirely preventable. To prevent it and ensure that the 2015 Sustainable Development Goals to reduce maternal mortality can be met, safe abortion care must be provided to the full extent of the law in ways that enable women to use the services they need.

## Data Availability Statement

The original contributions presented in the study are included in the article/Supplementary Material. Further inquiries can be directed to the corresponding author/s.

## Author Contributions

BD conceived the final research question and aims and objectives, reviewed the literature, and carried out the analysis. BD and DS jointly designed the study, devised the analysis strategy, and drafted the manuscript. Both authors read and approved the final manuscript.

## Conflict of Interest

The authors declare that the research was conducted in the absence of any commercial or financial relationships that could be construed as a potential conflict of interest.

## Publisher's Note

All claims expressed in this article are solely those of the authors and do not necessarily represent those of their affiliated organizations, or those of the publisher, the editors and the reviewers. Any product that may be evaluated in this article, or claim that may be made by its manufacturer, is not guaranteed or endorsed by the publisher.

## Abbreviations

CASP: critical appraisal skills programme
DRC: Democratic Republic of Congo
MMAT: mixed methods appraisal tool
MISP: Minimum Initial Service Package
NGO: non-governmental organisation
PRISMA-ScR: Preferred Reporting Items for Systematic reviews and Meta-Analyses extension for Scoping Reviews
SDGs: Sustainable Development Goals
SRH: sexual and reproductive health
ToP: termination of pregnancy
UN: United Nations
US: United States.
