# Supplementary material for: Maternal Health in Crisis: A Scoping Review of Barriers and Facilitators to Safe Abortion Care in Humanitarian Crises
Source: Front Glob Womens Health. 2021 Sep 21;2:699121. doi: 10.3389/fgwh.2021.699121 (PMC8594037; doi:10.3389/fgwh.2021.699121)
Supplement: Supplementary file 1 [file Table_1.docx]

**SUPPLEMENTARY MATERIALS**

**Supplementary material A**

*Search strategy on CINAHL PLUS: (03 July 2020)*

| **Search ID#** | **Search Terms** | **Results** |
| --- | --- | --- |
| S18 | S1 AND S4 AND S17 | 146 |
| S17 | S5 OR S6 OR S7 OR S8 OR S9 OR S10 OR S11 OR S12 OR S13 OR S14 OR S15 OR S16 | 573,346 |
| S16 | (((humanitarian or medical) W2 organi#ation*) or "MSF" or "Medecins Sans Frontieres" or "UNFPA" or "United Nations Population Fund" or "IFRC" or "Red Crescent" or "ICRC" or "Red Cross" or "IRC" or "International Rescue Committee" or "Marie Stopes" or "IPAS" or "Médicos Sem Fronteiras" or "Doctors Without Borders" or "Medicos Sin Fronteras" or "Fundo de População das Nações Unidas" or "Fondo de Población de las Naciones Unidas" or "Cruz vermelha" or "cruz roja") | 27,030 |
| S15 | nonprofit organizations | 5,276 |
| S14 | (MH "Organizations, Nonprofit+") | 30,823 |
| S13 | (MH "United Nations+") | 23,660 |
| S12 | (MH "International Agencies+") | 36,337 |
| S11 | (MH "Nursing Organizations, International+") | 5,683 |
| S10 | (MH "Nursing Organizations+") | 113,852 |
| S9 | (MH "American Red Cross") | 382 |
| S8 | American red cross | 0 |
| S7 | (MH "Medical Organizations+") | 37,864 |
| S6 | (MH "Red Cross+") | 1,338 |
| S5 | (MH "Organizations+") | 569,196 |
| S4 | S2 OR S3 | 12,310 |
| S3 | (abortion W2 (safe or comprehensive or induce* or provoke* or voluntary)) or (pregnancy W3 (end or terminat*))) | 12,150 |
| S2 | (MM "Abortion, Induced+") | 7,527 |
| S1 | implement* or provi* or deliver* or application or execut* or operationali?ation or reali?ation | 1,245,348 |

*Search strategy on EMBASE: (02 July 2020)*

| **Search ID#** | **Search Terms** | **Results** |
| --- | --- | --- |
| S1 | (implement* or provi* or deliver* or application or execut* or operationali#ation or reali#ation).mp. [mp=title, abstract, heading word, drug trade name, original title, device manufacturer, drug manufacturer, device trade name, keyword, floating subheading word, candidate term word] | 6,143,762 |
| S2 | ((abortion adj2 (safe or comprehensive or induce* or provoke* or voluntary)) or (pregnancy adj3 (end or terminat*))).mp. [mp=title, abstract, heading word, drug trade name, original title, device manufacturer, drug manufacturer, device trade name, keyword, floating subheading word, candidate term word] | 51,201 |
| S3 | exp abortion, induced/ | 37,863 |
| S4 | 2 or 3 | 62,926 |
| S5 | (((humanitarian or medical) adj2 organi#ation*) or "MSF" or "Medecins Sans Frontieres" or "Médicos Sem Fronteiras" or "Doctors Without Borders" or "Medicos Sin Fronteras" or "UNFPA" or "United Nations Population Fund" or "Fundo de População das Nações Unidas" or "Fondo de Población de las Naciones Unidas" or "IFRC" or "Red Crescent" or "ICRC" or "Red Cross" or "Cruz vermelha" or "cruz roja" or "IRC" or "International Rescue Committee" or "Marie Stopes" or "IPAS").mp. [mp=title, abstract, heading word, drug trade name, original title, device manufacturer, drug manufacturer, device trade name, keyword, floating subheading word, candidate term word] | 18,598 |
| S6 | exp organizations/ | 766,064 |
| S7 | exp red cross/ | 4,331 |
| S8 | exp relief work/ | 1,345 |
| S9 | exp united nations/ | 119,035 |
| S10 | exp organizations, nonprofit/ | 36,676 |
| S11 | 5 or 6 or 7 or 8 or 9 or 10 | 810,627 |
| S12 | 1 and 4 and 11 | 1,332 |
| S13 | limit 12 to (abstracts and embase and (english or portuguese or spanish) and  yr="2010 - 2020") | 274 |

*Search strategy on OVID GLOBAL HEALTH: (03 July 2020)*

| **Search ID#** | **Search Terms** | **Results** |
| --- | --- | --- |
| S1 | (implement* or provi* or deliver* or application or execut* or operationali#ation or reali#ation).mp. [mp=abstract, title, original title, broad terms, heading words, identifiers, cabicodes] | 775,069 |
| S2 | ((abortion adj2 (safe or comprehensive or induce* or provoke* or voluntary)) or (pregnancy adj3 (end or terminat*))).mp. [mp=abstract, title, original title, broad terms, heading words, identifiers, cabicodes] | 4,460 |
| S3 | exp abortion/ | 9,878 |
| S4 | 2 or 3 | 12,937 |
| S5 | (((humanitarian or medical) adj2 organi#ation*) or "MSF" or "Medecins Sans  Frontieres" or "Médicos Sem Fronteiras" or "Doctors Without Borders" or  "Medicos Sin Fronteras" or "UNFPA" or "United Nations Population Fund" or  "Fundo de População das Nações Unidas" or "Fondo de Población de las  Naciones Unidas" or "IFRC" or "Red Crescent" or "ICRC" or "Red Cross" or  "Cruz vermelha" or "cruz roja" or "IRC" or "International Rescue Committee" or  "Marie Stopes" or "IPAS").mp. [mp=abstract, title, original title, broad terms,  heading words, identifiers, cabicodes] | 2,954 |
| S6 | exp organizations/ | 38,530 |
| S7 | exp united nations/ | 10,065 |
| S8 | 5 or 6 or 7 | 41,258 |
| S9 | 1 and 4 and 8 | 99 |
| S10 | limit 9 to (abstracts and (english or portuguese or spanish) and yr="2010 - 2020") | 59 |

*Search strategy on OVID MEDLINE: (04 July 2020)*

| **Search ID#** | **Search Terms** | **Results** |
| --- | --- | --- |
| S1 | (implement* or provi* or deliver* or application or execut* or operationali#ation or reali#ation).mp. [mp=title, abstract, original title, name of substance word, subject heading word, floating sub-heading word, keyword heading word, organism supplementary concept word, protocol supplementary concept word, rare disease supplementary concept word, unique identifier, synonyms] | 3,901,108 |
| S2 | ((abortion adj2 (safe or comprehensive or induce* or provoke* or voluntary)) or (pregnancy adj3 (end or terminat*))).mp. [mp=title, abstract, original title, name of substance word, subject heading word, floating sub-heading word, keyword heading word, organism supplementary concept word, protocol supplementary concept word, rare disease supplementary concept word, unique identifier, synonyms] | 40,348 |
| S3 | exp abortion, induced/ | 40,100 |
| S4 | 2 or 3 | 50,070 |
| S5 | (((humanitarian or medical) adj2 organi#ation*) or "MSF" or "Medecins Sans  Frontieres" or "Médicos Sem Fronteiras" or "Doctors Without Borders" or  "Medicos Sin Fronteras" or "UNFPA" or "United Nations Population Fund" or  "Fundo de População das Nações Unidas" or "Fondo de Población de las  Naciones Unidas" or "IFRC" or "Red Crescent" or "ICRC" or "Red Cross" or  "Cruz vermelha" or "cruz roja" or "IRC" or "International Rescue Committee" or  "Marie Stopes" or "IPAS").mp. [mp=title, abstract, original title, name of  substance word, subject heading word, floating sub-heading word, keyword  heading word, organism supplementary concept word, protocol supplementary  concept word, rare disease supplementary concept word, unique identifier,  synonyms] | 11,097 |
| S6 | exp organizations/ | 452,644 |
| S7 | exp red cross/ | 2,227 |
| S8 | exp relief work/ | 5,060 |
| S9 | exp united nations/ | 40,917 |
| S10 | exp organizations, nonprofit/ | 19,338 |
| S11 | 5 or 6 or 7 or 8 or 9 or 10 | 463,977 |
| S12 | 1 and 4 and 11 | 633 |
| S13 | limit 12 to (abstracts and yr="2010 - 2020" and (english or portuguese or spanish)) | 136 |

*Search strategy on RELIEFWEB: (04 July 2020)*

| **Search ID#** | **Search Terms (“Any of”)** | |
| --- | --- | --- |
| S1 | "safe abortion" OR "termination of pregnancy" OR "comprehensive abortion" OR "induced abortion" OR "voluntary abortion" OR "provoked abortion" OR "terminate pregnancy" OR "TPR" | AND |
| S2 | Organization type: International organization  Organization type: Non-governmental organization  Organization type: Red Cross/Red Crescent Movement  Organization type: Other | AND |
| S3 | Language: English  Language: Spanish | AND |
| S4 | Original publication date: 2010/01/01 to 2020/01/01 | 57 results |

*Search strategy on CONFLICT AND HEALTH JOURNAL: (04 July 2020)*

| **Search term** | **Results** |
| --- | --- |
| Safe abortion | 38 |
| Termination of pregnancy | 10 |
| Induced abortion | 13 |
| **Total** | **61** |

*Search strategy on HEALTH AND HUMAN RIGHTS JOURNAL: (04 July 2020)*

| **Search term** | **Results** |
| --- | --- |
| Safe abortion | 107 |
| **Total** | **107** |

**Supplementary material B**

***Complete table of findings***

| **Refe-rence** | **Authors, Year of Publication** | **Region** | **Source type** | **Research design** | **Population** | **Barriers to safe abortion care** | **Facilitators to safe abortion care** |
| --- | --- | --- | --- | --- | --- | --- | --- |
| 15 | Myers et al, 2018 | Nepal | Research (Conflict and Health) | Mixed methods | 249 adults aged 18 – 49; 26 key informants; and 17 health facilities (assessments) | 1. Geographical barriers  2. Health facilities destruction  3. Inadequate staffing  4. Poor SRH level of coordinators and managers  5. External support unwelcomed by communities  6. High costs  7. Intermittent funding  8. Logistical challenges  9. Low awareness of existing local capacity  10. Prioritization of basic needs instead of SRH  11. Low reporting  12. Stigma  13. Under-resourced facilities in rural areas  14. Weak communication between stakeholders | 1. Disaster preparedness  2. Leadership of organizations  3. Resource mobilization  4. National level coordination  5. Existing SRH and outreach programs  6. Supply chain management  7. Established pre-crisis health services  8. Pre-established Memorandum of Understanding between Ministry of Health and NGOs |
| 29 | Stifani et al, 2018 | Colombia | Research (CINAHL Plus) | Qualitative | 13 key stakeholders and experts | 1. Bureaucracy  2. Conscientious objection  3. Geographical barriers  4. Lack of quality audit  5. Lack of ToP provision  6. Lack of legal knowledge  7. Long waiting periods  8. Restrictive abortion law  9. Stigma | 1. NGOs included in the ToP services rollout  2. Ministry of Health support  3. ToP included in general SRH care  4. Low tech ToP options  5. Political opportunity window (political willingness) |
| 42 | Chynoweth, 2015 | Worldwide | Practice literature (Conflict and Health) | Practice literature | 4 complementary studies done as part of the IAWG global evaluation in 2014 | 1. Exclusion of ToP topic | None related to ToP |
| 23 | Palmer & Storeng, 2016 | South Sudan | Research (Elsevier) | Qualitative | 54 Key informants | 1. Security risk (patients and staff)  2. International NGOs not allowed to work in the country  3. Donors opposed to ToP provision  4. Patriarchal society  5. Restrictive abortion law  6. Suspicion related to family planning  7. Women as biological & cultural ‘transmitters’  8. Spousal’s consent required for contraception | 1. Political opportunity window (end of war)  2. Stakeholders involvement in workshops  3. International NGO-led or private SRH clinics  4. ToP and contraceptives offered in secret as individual harm-reduction  5. International NGOs involved in health policy formulation |
| 27 | Tran et al, 2015 | Worldwide | Research (PubMed) | Mixed-Methods | 82 institution representatives from 48 countries | 1. Lack of ToP and port-abortion care provision and referrals | 1. Growth of SRH-related institutional capacity |
| 43 | Casey, 2015 | Worldwide | Research (Conflict & Health) | Systematic review | 36 papers describing 30 programs | 1. Exclusion of ToP and port-abortion care from the programs | None described |
| 30 | Nara et al, 2019 | Uganda | Research (Embase) | Qualitative | 11 key informants; 36 Congolese women through 4 FGD; and 21 in-depth interviews w/ congolese women of reproductive age | 1. Poor port-abortion care seeking behaviour  2. Security risk (staff and patients)  3. High costs  4. Lack of legal knowledge  5. Restrictive abortion law | 1. Port-abortion care provision  2. Port-abortion care not legally restricted |
| 26 | Banwell, 2020 | Worldwide | Practice literature (Taylor & Francis Online) | Practice literature | n/a | 1. Lack of funding  2. Lack of ToP provision | None described |
| 31 | Casey et al, 2015 | Burkina Faso, Democratic Republic of Congo (DRC) and South Sudan | Research (Conflict and Health) | Mixed-Methods | 63 health facilities (assessments); 42 healthcare providers; 29 members of the host communities; and 273 displaced people | 1. Health facilities unauthorized to provide ToP  2. Lack of ToP provision  3. Lack of supplies  4. Lack of trained staff  5. Health centres did not meet port-abortion care requirements  6. Stigma  7. Religious beliefs against ToP | 1. All hospitals met port-abortion care requirements  2. ToP permitted under certain circumstances |
| 32 | Radhakrishnan et al, 2017 | Worldwide | Practice literature (Medline) | Practice literature | n/a | 1. Lack of funding  2. Lack of ToP provision  3. Restrictive abortion law | 1. IHL  2. Segregation of humanitarian aid from US donors to avoid aid restrictions  3. US Leahy Amendment permits counselling and information about all pregnancy options |
| 39 | Steven et al, 2019 | DRC | Research (CINAHL Plus) | Qualitative | 12 key informant interviews with men | 1. Pro-natalist tradition  2. Inadequate SRH infrastructure  3. Violence  4. Patriarchal society | 1. Community leaders as agents of change  2. Engagement between INGOs and community leaders |
| 25 | Krause et al, 2015 | Jordan | Research (Conflict and Health) | Mixed-methods | 11key informants; 12 health faciliies (assessments); and 159 women through 14 FGDs | 1. Lack of protocol  2. Restricted contraceptives  3. Lack of female SRH staff  4. Lack of funding  5. Lack of awareness on free SRH services  6. Lack of supplies  7. Overloaded health system  8. Perceived bad quality of SRH services  9. Stigma | 1. Available SRH funding  2. Skilled work force  3. Leadership of organizations  4. Existing SRH programs |
| 44 | Whitmill et al, 2016 | Worldwide | Research (PubMed) | Quantitative | 10 refugee camps managed by UNHCR | 1. Exclusion of ToP and/or port-abortion care topics from the list of SRH indicators | None described |
| 47 | Foster et al, 2017 | Worldwide | Practice literature (Medline) | Practice literature | n/a | None described | 1. The inclusion of a ToP chapter in the manual is likely to impact the prioritization of preventing and treating unintended pregnancies |
| 24 | Shahawy, 2019 | Occupied Palestinian Territories | Research (Health & Human Rights) | Qualitative | 60 Palestinian women in East Jerusalem | 1. Suspicion related to family planning services  2. Security risk  2. Fragmented nature of health system  3. High costs  4. Spousal’s consent required for SRH services  5. Forced travel to access ToP  6. Pro-natalist traditions  7. Travel restrictions  8. Restrictive abortion law  9. Restrictive hospital ToP policies  10. Stigma | 1. ToP permitted under certain circumstances |
| 46 | Tanabe et al, 2015 | Worldwide | Practice literature (Conflict and Health) | Quantitative | n/a | 1. Abortion was rarely mentioned  2. Smallest share of SRH funding to family planning and port-abortion care  3. Low number of family planning funding proposals  4. Lack of funding  5. Exclusion of ToP topic | 1. Increased SRH funding  2. Increased number of SRH proposals classified as MISP  3. Increase in the number of SRH proposals compared to previous years |
| 41 | Patel et al, 2016 | Worldwide | Research (Wiley) | Quantitative | 18 conflict-affected countries that receive official development assistance (ODA) disbursement for RH activities | 1. Aid disbursed for ToP could not be disaggregated from the general SRH care  2. Conflict-affected countries received less funding for SRH than non-conflict-affected countries, even when the needs were higher | 1. Increased SRH funding over the 10 past years |
| 5 | McGinn & Casey, 2016 | Worldwide | Practice literature (Conflict and Health) | Practice literature | n/a | International NGOs claim that:  1. It is too complicated to provide ToP  2. Donors do not fund ToP  3. Illegality of ToP in the country of operations  4. No need to provide ToP | 1. Low-tech ToP options  2. Non-US donors fund ToP  3. In 190 countries ToP is permitted under some circumstances  4. ToP as a justified need due to the collapse of the health system and unwanted pregnancies  5. ToP among the safest medical procedures  6. International Humatirian Law |
| 33 | Schulte-Hillen et al, 2016 | Worldwide | Practice literature (Conflict and Health) | Practice literature | n/a | 1. Negative community perception (MSF taking ToP business from local providers)  2. Difficulty to navigate through legal restrictions  3. High turnover of international staff  4. NGO internal resistance  5. Lack of staff commitment  6. Lack of legal abortion knowledge  7. Conscientious objection  8. Security risk  10. Tension with local authorities | 1. Confidentiality  2. International staff to assume the responsibility of providing ToP  3. Back up of professionals providing ToP  4. Inclusion of ToP in obstetric guidelines  5. To create an environment in which personal feelings towards ToP can co-exist the professional responsibility  6. Private donations to ensure independence of operations  7. Referral to ToP when other actors present  8. Key stakeholders’ networking  9. Clear ToP policy  10. Sensitization of medical staff |
| 40 | Doctors Without Borders, 2020 | Worldwide | Practice literature (Conflict and Health) | Practice literature | n/a | 1. Lack of funding  2. Closure of NGOs  3. Stigma  4. Increased unsafe abortions worldwide | None described |
| 38 | Tran & Schulte-Hillen, 2018 | Worldwide | Practice literature (Conflict and Health) | Practice Literature | n/a | 1. At the onset of a humanitarian crises ToP should not be part of the MISP as it might put field staff, patients and operations at risk | None described |
| 34 | Tousaw et al, 2017 | Thailand | Research (Medline) | Qualitative | 22 women who sought safe abortion through the Safe Abortion Referral Programme (SARP) | 1. Restrictive abortion law  2. Lack of documentation of refugees  3. Lack of ToP provision  4. Language barriers to foreigners  5. Lack of knowledge on ToP  6. Lack of awareness on free SRH services  7. Lack of legal abortion knowledge  8. Financial insecurity  10. High costs  11. Travel restrictions  12. Stigma  13. Discrimination  14. Eligibility criteria  15. Lack of funding | 1. Financial support  2. Positive perception of beneficiaries  3. Community engagement  4. Counsellors were trusted community members  5. Counsellors participated in a training prior to the programme's launch  5. Community awareness of the services  6. After using the program, women became advocates for ToP and SRH |
| 45 | Edwards G, 2017 | Worldwide | Practice literature (CINAHL Plus) | Practice literature | n/a | 1. Lack of contraceptive provision  2. Lack of funding | None described |
| 35 | Casey et al, 2019 | DRC | Research (Medline) | Qualitative | 246 adults aged 18 to 45 years old from the rural health zones of North and South Kivu | 1. Collapse of health system  2. Conflict-related SV  3. High costs  4. Lack of research on abortion stigma and evidence-based interventions to address negative beliefs towards ToP in low and middle income countries  5. Lack of trained healthcare providers  6. Restrictive abortion law  7. Lack of supply  8. Stigma  9. Spousal/ familial abandonment; exclusion by the community | 1. Community engagement  2. Signature and ratification of the Maputo Protocol  3. Political opportunity window |

**Supplementary material C**

***MMAT for Qualitative Research***

| **Author/Year**  **(Study)** | **Are there clear research questions?** | **Do the collected data allow to address the research questions?** | **1.1. Is the qualitative approach appropriate to answer the research question?** | **1.2. Are the qualitative data collection methods adequate to address the research question?** | **1.3. Are the findings adequately derived from the data?** | **1.4. Is the interpretation of results sufficiently substantiated by data?** | **1.5. Is there coherence between qualitative data sources, collection, analysis and interpretation?** | **Score** |
| --- | --- | --- | --- | --- | --- | --- | --- | --- |
| Stifani et al, 2018 | Yes | Yes | Yes | Unclear | Yes | Yes | Yes | 80% |
| Palmer & Storeng, 2016 | Yes | Unclear | Yes | Unclear | No | Unclear | Yes | 40% |
| Nara, Banura & Foster, 2019 | Yes | Yes | Yes | Yes | Yes | Yes | Yes | 100% |
| Steven et al, 2019 | Yes | Yes | Yes | Yes | Yes | Yes | Yes | 100% |
| Shahawy, 2019 | Yes | Unclear | Yes | Unclear | Unclear | No | Yes | 40% |
| Tousaw et al, 2017 | Yes | Yes | Yes | Yes | Yes | Yes | Yes | 100% |
| Casey et al, 2019 | Yes | Yes | Yes | Yes | Yes | Yes | Yes | 100% |

***MMAT for Quantitative Research***

| **Author/Year**  **(Study)** | **Are there clear research questions?** | **Do the collected data allow to address the research questions?** | **4.1. Is the sampling strategy relevant to address the research question?** | **4.2. Is the sample representative of the target population?** | **4.3. Are the measurements appropriate?** | **4.4. Is the risk of nonresponse bias low?** | **4.5. Is the statistical analysis appropriate to answer the research question?** | **Score** |
| --- | --- | --- | --- | --- | --- | --- | --- | --- |
| Whitmill et al, 2016 | Yes | Yes | Yes | No | Yes | No | Yes | 60% |
| Patel et al, 2015 | Yes | Yes | Yes | Yes | Yes | Yes | Yes | 100% |

***MMAT for Mixed-Methods Research***

| **Author/Year**  **(Study)** | **Are there clear research questions?** | **Do the collected data allow to address the research questions?** | **4.1. Is the sampling strategy relevant to address the research question?** | **4.2. Is the sample representative of the target population?** | **4.3. Are the measurements appropriate?** | **4.4. Is the risk of nonresponse bias low?** | **4.5. Is the statistical analysis appropriate to answer the research question?** | **Score** |
| --- | --- | --- | --- | --- | --- | --- | --- | --- |
| Myers et al, 2015 | Yes | Yes | Yes | Yes | Unclear | No | Yes | 60% |
| Krause et al, 2015 | Yes | Yes | Yes | Unclear | No | Unclear | No | 20% |
| Casey et al, 2015 | Yes | Yes | Yes | Yes | Yes | Yes | Yes | 100% |
| Tran et al, 2015 | Yes | Yes | Yes | Unclear | Unclear | Unclear | Unclear | 20% |

**Supplementary material D**

**Aveyard et al (2015) appraisal tool**

| **Author, year** | **Source of information is reliable?** | **Is it a research paper?** | **Is it of good quality? (Is their line of reasoning logical and understandable?)** | **Author/Organization is an expert in field?** | **Published in 2015 or later?** | **Information aimed at professionals?** | **Score** |
| --- | --- | --- | --- | --- | --- | --- | --- |
| Foster et al, 2017 | Partly (PubMed but not research) | No (Practice literature) | Partly (Secondary research) | Yes | Yes | Yes | 60% |
| Doctors Without Borders, 2018 | Partly (NGO website, not research) | No (Practice literature) | Yes | Yes | Yes | No | 60% |
| Schulte-Hillen, Staderini & Saint-Sauveur, 2016 | Partly (PubMed but not research) | No (Practice literature) | Yes | Yes | Yes | Yes | 70% |
| McGinn & Casey, 2016 | Partly (Conflict & Health, but not research) | No (Practice literature) | Yes | Yes | Yes | Yes | 70% |
| Banwell, 2020 | Partly (TandF, but not research) | No (Practice literature) | No | Partly | Yes | Yes | 40% |
| Radhakrishnan, Sarver & Shubin, 2017 | Partly (TandF, but not research) | No (Practice literature) | Yes | Yes | Yes | Yes | 70% |
| Tanabe et al, 2015 | Partly (Conflict & Health, but not research) | No (Practice literature) | Yes | Yes | Yes | Yes | 70% |
| Chynoweth, 2015 | Partly (Conflict & Health, but not research) | No (Practice literature) | Partly | Yes | Yes | Yes | 60% |
| Edwards, 2017 | Partly (NGO website but anedoctal information) | No (Practice literature) | Yes | Yes | Yes | No | 60% |
| Toan Tran & Schulte-Hillen, 2018 | Partly (Conflict & Health, but not research) | No (Practice literature) | Yes | Yes | Yes | Yes | 70% |

**Supplementary material E**

***CASP Appraisal***

| **Casey, 2015** | **Yes** | **No** | **Can’t Tell** |
| --- | --- | --- | --- |
| Did the review address a clearly focused question? | x |  |  |
| Did the authors look for the right type of papers? | x |  |  |
| Do you think all the important, relevant studies were included? | x |  |  |
| Did the review’s authors do enough to assess quality of the included studies? | x |  |  |
| If the results of the review have been combined, was it reasonable to do so? | x |  |  |
| What are the overall results of the review? | Only1 out of the 29 studies selected included ToP and this was the main finding: after investing on HR and supply, a hospital in Sierra Leone saw the proportion of women accessing it from 31 in 1990 to 98 in 1995, with a reduction in the case-fatality rate from 32% to 5%. Also, 444 induced abortion-related procedures were performed, compared with only 22 in 1990 | | |
| How precise are the results? | Precise | | |
| Can the results be applied to the local population? | x |  |  |
| Were all important outcomes considered? |  | x |  |
| Are the benefits worth the harms and costs? | x |  |  |
| **Score** | 90% | | |
